# A light-guiding urinary catheter for the inhibition of *Proteus mirabilis* biofilm formation

**DOI:** 10.3389/fmicb.2022.995200

**Published:** 2022-09-20

**Authors:** Jonathan T. Butement, Daniel J. Noel, Catherine A. Bryant, Sandra A. Wilks, Robert W. Eason

**Affiliations:** ^1^Optoelectronics Research Centre, University of Southampton, Southampton, United Kingdom; ^2^School of Biological Sciences, University of Southampton, Southampton, United Kingdom; ^3^School of Health Sciences, University of Southampton, Southampton, United Kingdom

**Keywords:** biofilms, photonics, antimicrobials, blue light, catheter associated urinary tract infection

## Abstract

Catheter-associated urinary tract infection (CAUTI) is a leading cause of hospital-acquired infections worldwide causing debilitating illness for patients as well as a significant financial and treatment burden on health services. CAUTI is linked with the build-up of biofilms on catheter surfaces which act as a reservoir for infection. Additionally, urease-producing bacteria such as Gram-negative *Proteus mirabilis* (*PM*), can form crystalline biofilms which encrust catheter surfaces ultimately leading to blockages which require immediate removal of the catheter. Currently there are limited treatments available to prevent the formation of biofilms by *PM* as well as other urinary tract infection causing bacteria. A novel concept for a light-guiding urinary catheter is presented where a silicone elastomer waveguide incorporated along the length of the catheter is used to irradiate the catheter surfaces with antimicrobial blue light (405 nm) to prevent biofilm formation *in situ*. The prototype device is mass producible while also easy to fabricate in a lab setting for research studies. The inhibitory effect of blue light on *PM* biofilm formation over a range of irradiances is described for the first time showing an LD_90_ at 192–345 J/cm^2^ and total inhibition at 1,700 J/cm^2^ *In vitro* studies show that the light-guiding catheter (LGC) prototypes exhibit a 98% inhibition in *PM* biofilm formation inside the catheter lumen at an average estimated irradiance of 30–50 mW/cm^2^ (324–540 J/cm^2^ fluence) showing that the concept is highly effective, promising to be a powerful and economical antimicrobial approach to prevent catheter associated biofilm development and blockage.

## Introduction

Catheter-associated urinary tract infections (CAUTIs) account for 45–68% of hospital-acquired UTIs in the UK and US. At any one time around 1 in 5 hospitalized patients has an indwelling urinary catheter (Magill et al., 2014; Public Health England, 2016) which in English NHS hospitals alone equates to approximately 1M patients per year (Smith et al., 2019). The frequency of use of catheters and increased risk of secondary bloodstream infection makes CAUTI a significant cause of morbidity and mortality. In hospitals and care homes, indwelling urinary catheters comprise an important institutional reservoir of pathogenic bacteria, including antimicrobial-resistant strains (Maki and Tambyah, 2001). Indwelling urinary catheters increase the risk of UTI as bacteria from outside the body may be introduced into the urinary tract and form resilient, structured colonies on catheter surfaces known as biofilms which then act as a reservoir for infection. Significantly, biofilm growth inside catheters can also lead to blockage of flow causing an acute medical emergency which requires immediate replacement of the catheter. One bacterium, involved in 10–44% of long term CAUTIs in the United States (Schaffer and Pearson, 2015), and particularly implicated in catheter blockage is *Proteus mirabilis* (*PM*). It forms crystalline biofilms due to urease activity which increases the pH of urine leading to mineral precipitation as crystals causing encrustations on the internal surfaces of a catheter which occlude flow (Morris and Stickler, 1998; Jones et al., 2005). The risk of catheter blockage increases with duration of catheter use, resulting in additional risk for long term users. Blockages can have severe implications for users and place a high burden on healthcare providers. *PM* is also motil*e* (Armbruster and Mobley, 2012) allowing it to propagate along catheter surfaces further into the urinary tract. Currently there are limited therapeutic strategies available for inhibiting PM biofilm formation (Wasfi et al., 2020) and so there is pressing requirement for new antimicrobial approaches targeted at PM.

A range of approaches have been developed to prevent biofilm build-up on catheters but with limited efficacy and significant practical limitations. Antimicrobial coatings such as silver alloys, nitrofurazone and antibiotics have shown minimal effect on biofilm adherence and limited clinical efficacy in preventing CAUTI (Desai et al., 2010; Al-Qahtani et al., 2019). Catheter silicone embedded with a combination of a photosensitizer, methylene blue, and gold nanoparticles and irradiated by laser light to produce reactive species was shown to reduce catheter material biofilm coverage by 50% (Perni et al., 2010). The major drawback of coated catheters and photosensitizer coatings is the physical properties of biofilms prevent access of the antimicrobials to the target cells, as well as potential for side effects from leaching catheter materials. The bacteria within the biofilm are encased in a self-produced matrix of extra-polymeric substances (EPS) providing increased tolerance to the cells. Ultrasonic waves transmitted down the length of the catheter have shown some effectiveness, however, the equipment is bulky, expensive and prone to disruption by movement (Wang H. et al., 2017).

New effective technologies are needed to prevent biofilm formation on catheters to lower the risk of blockages and CAUTI, reduce antibiotic use and alleviate the associated financial burden to health services. The use of blue light as an antimicrobial agent has attracted considerable attention (Wang Y. et al., 2017; Gwynne and Gallagher, 2018; Leanse et al., 2022). It is cost-effective, selectively inhibits bacterial cells, does not require additional photosensitizers and is a means to overcome antimicrobial resistance (AMR). The antimicrobial activity of blue light has been demonstrated to excite naturally occurring porphyrins and/or flavins within the microbial cell (Ashkenazi et al., 2003; Cieplik et al., 2014), which promote the synthesis of a range of cytotoxic oxidative species primarily singlet oxygen (O_2_) (Cieplik et al., 2014). While all wavelengths from 400 to 470 nm can be used for microbial inactivation the optimal antimicrobial activity occurs between 400 and 405 nm. Importantly, blue light therapy is effective in the inactivation of a wide range of key bacterial and fungal pathogens (Halstead et al., 2016) irrespective of their antimicrobial-resistance properties. Significantly, blue light is efficacious for the inactivation of both planktonic cells and those formed within a biofilm. Unlike sub-lethal antimicrobial drug exposure, blue light exposure has shown no development of resistance in Gram-positive (Tomb et al., 2017) and Gram-negative bacteria (Leanse et al., 2018). Blue light has also been shown to inhibit bacteria responsible for CAUTIs (Halstead et al., 2016). Blue light is appealing for use in indwelling medical devices such as urinary catheters as it is less damaging to human tissue than UV light while still being bactericidal. At high dosage (fluence) blue light is toxic to human cells through the production of reactive oxygen species (Liebmann et al., 2010; Ramakrishnan et al., 2016; Wang et al., 2020), however, the toxic effect of blue light on human cells can be minimized by moderating dose; for example by LED pulsing (Gillespie et al., 2017), or using blocking structures in biomedical devices to protect sensitive tissues. The use of antimicrobial blue light has been suggested for use in catheters, however, there have only been a few attempts at creating viable devices with limited testing of antimicrobial efficacy (Klubben et al., 2016; Vollmerhausen et al., 2017). Additionally there has been limited demonstration of the effect of blue light on *PM* viability (Ferrer-Espada et al., 2020; Funjan, 2021).

For antimicrobial blue light to be effective it must be delivered efficiently to catheter surfaces. The lumen is a primary target as it is prone to biofilm growth which can cause blockages or eventually infection of the bladder. Light-guides, including optical fibers and integrated waveguides, are an ideal technology for delivering the light to biofilm-susceptible surfaces. These micrometer to millimeter scale structures are easy to integrate into tubular catheter designs and allow bulky light sources with any associated electrical connections and power supply to remain outside of the body while the antimicrobial light is transmitted efficiently to catheter surfaces *in situ.*

### Light-guiding catheter concept

**FIGURE 1:**
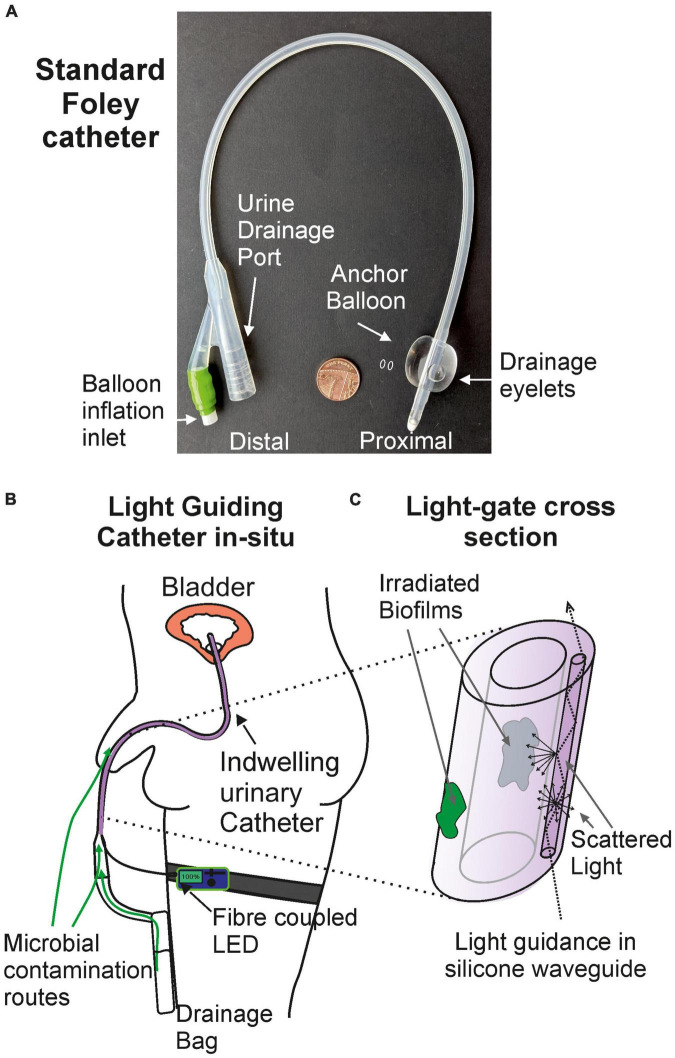
The light-guiding catheter concept. **(A)** Photograph of a standard silicone foley catheter. **(B)** Light-guiding catheter *in situ*. **(C)** Cross section of the antimicrobial light gate section of the light-guiding catheter and the mechanism of biofilm irradiation.

In this work we propose the use of a light-guiding catheter (LGC) to deliver antimicrobial blue light to catheter surfaces *in situ* thus inhibiting the formation of biofilms. The device design is modeled on a standard silicone foley catheter shown in Figure 1A for compatibility with standard catheter manufacturing practices such as extrusion and injection molding. Figure 1B shows the LGC concept where an LED on a leg strap is connected *via* an optical fiber to a leaky waveguide which is embedded in the side wall of the catheter running all the way up to the inflation balloon at the distal end. The waveguide core is made of optical grade silicone with a higher refractive index (e.g., refractive index contrast Δ = 0.037) than the surrounding bulk catheter silicone. Light is guided by total internal reflection along the length of the catheter, partly in the waveguide core and partly in the catheter side wall, with a proportion of light being scattered sideways irradiating the internal and external catheter surfaces. In the device presented here, scattering is caused by surface roughness at the interface between the waveguide core and cladding as well as by fumed silica particles inside the waveguide core of contrasting refractive index to the waveguide core material. The result is a side illumination profile with decreasing irradiance from the proximal to the distal end of the catheter. The higher side irradiance at the distal portion of the catheter forms an antimicrobial “light gate” which inhibits the formation of biofilms in this region and prevents ingress of bacteria along catheter surfaces further into the urinary tract.

The use of thermoset optical silicone potentially allows the distribution of scattering particles in the waveguide core to be controlled during injection molding so that the scattering profile along the length of the waveguide can be specifically controlled. Crucially the silicone elastomer forming the waveguide core can stretch up to three times its own length and so the longitudinal elasticity of the catheter is preserved which is vital for patient comfort and to reduce the risk of internal injury caused by tugging on the catheter. Alternative LGC designs use inextensible (glass) optical fibers which run the length of the catheter and compromise the catheter elasticity (Eltorai, 2019) or metallic films which can be broken by stretching of the catheter (Rhodes et al., 2017). The LGC is economical as the optical silicone structures and connecting fibers only add a marginal cost to the disposable catheter unit and the reusable LED package is of comparable cost to a bicycle light. A typical 405 nm LED with an energy efficient design drawing 250 mA will run for at least 24 h between battery changes on an 8,000 mAh battery unit weighing 50 g.

In this study, the antimicrobial effect of blue light irradiance on *PM* biofilm formation on silicone is first evaluated followed by a demonstration of the antimicrobial efficacy of the LGC device in an *in vitro* model study with *PM*.

## Results and discussion

### Fabrication and optical characterization of light-guiding catheters

**FIGURE 2:**
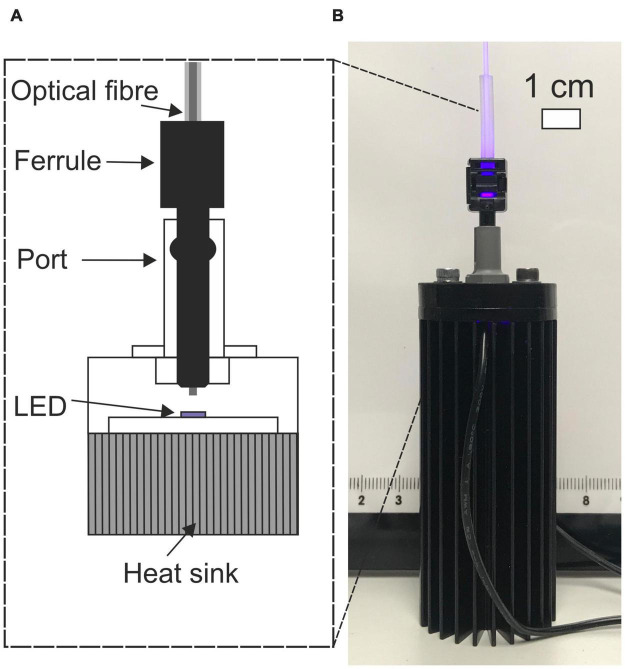
**(A)** Cut-away schematic of LED-fiber butt-coupling assembly. **(B)** Photograph of fiber coupled LED.

The prototype light guiding catheter systems were designed with off the shelf components for accessibility. The injection molding approach for producing waveguides was very convenient for rapid prototyping and could be easily reproduced in any microbiology laboratory or upscaled for mass production. Six complete optical systems comprising the LED, coupling optical fiber and LGC were optically characterized. The LED-fiber butt-coupling assembly is shown in Figure 2. The maximum output power from the optical fibers was measured at 72 ± 8 mW giving an LED-to-optical fiber coupling loss of 9.7 ± 0.5 dB. Coupling losses were high in this case as the PMMA fiber had to be backed off from the LED surface to prevent melting. Using a high numerical aperture glass fiber would allow contact coupling to the LED surface adding some cost to the system but improving optical efficiency. These lab-made LED modules were of comparable optical efficiency to fiber coupled LEDs produced by commercial suppliers, at one tenth of the cost making the units more accessible for upscaled research.

**FIGURE 3:**
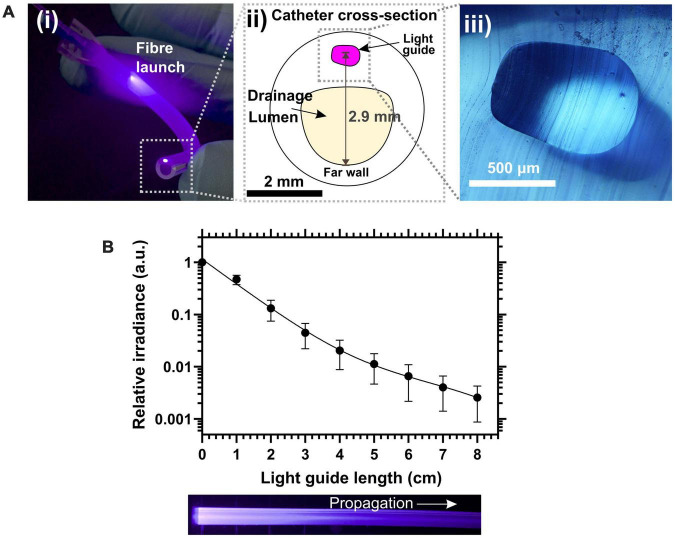
**(A)** (i) Photograph of a 5 cm length of light-guiding catheter showing the fiber launch position and the illuminated spot from the waveguide core at the severed upstream end of the catheter. (ii) Schematic of the cross section of the light-guiding catheter. (iii) Micrograph of the silicone waveguide core from a slice of light-guiding catheter. **(B)** (Top) Relative side emission irradiance along catheter length. (Bottom) Photograph in plain view of the waveguide side of the catheter.

The coupling loss between the input optical fiber and the waveguide was within a range of 4.13–6.36 dB. The photograph in Figure 3Ai shows a 5 cm section of LGC held in hand. At the region where the fiber launches light into the waveguide, excess light can be seen leaking into the bulk of the catheter because the 1 mm diameter fiber is wider than the waveguide and has a higher numerical aperture of 0.5 compared to 0.41 for the waveguide. Light leakage is also due to the mismatch between the circular fiber cross section and the asymmetric shape of the light guide which is shown in Figures 3Aii,iii and was dictated by the original shape of the balloon inflation channel. The excess leaking light is coupled into the sidewall of the catheter tubing which itself acts as a leaky waveguide transmitting light along the length of the catheter. The combined effect of the leaking light and the light scattered from the waveguide form the antimicrobial light gate which extends with decreasing irradiance from the drainage (proximal) end of the catheter toward the bladder (distal) end. Future LGC catheter designs will aim to minimize coupling loss into the waveguide by matching the geometry and numerical aperture to the input optical fiber. The propagation loss for the waveguide was measured by cut-back at 1.3 ± 0.4 dB/cm. Figure 3B shows the experimentally measured decay profile of the irradiance from the side emission from the waveguide along the length of the catheter which follows a double exponential decay profile. The overall optical performance of the LGC devices produced, considering they were adapted from standard catheters, was deemed to be sufficient for use in microbiological efficacy tests.

### Antimicrobial effect of blue light irradiance on *Proteus mirabilis*

For the antimicrobial light gate of the LGC to work, the irradiance inside the catheter must be high enough to provide a significant antimicrobial effect over a short period of time to prevent *PM* biofilms from forming on catheter surfaces or moving past the irradiated section of the catheter (Belas et al., 1998; Manos et al., 2004). Our control experiments showed that significant seeding of biofilms on catheter surfaces from contaminated artificial urine occurred over a short time of 180 min in the absence of blue light. To determine an effective antimicrobial range of irradiance over this short period, an experiment was conducted with irradiance as the independent variable. Silicone coupons were incubated in the presence of *PM*-spiked artificial urine under a range of irradiances over 180 min and the extent of biofilm formation was compared.

**FIGURE 4:**
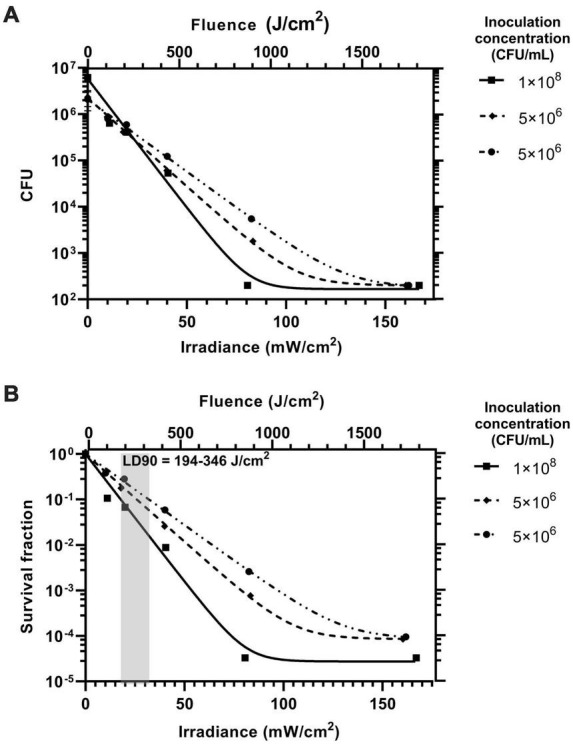
The inhibitory effect of blue light irradiance on the formation of *Proteus mirabilis* biofilms on catheter silicone represented on a logarithmic scale by **(A)** CFU and **(B)** survival fraction. The inoculation concentration of the AUM for each of the three experiments is displayed in the legend. The fluence for the exposure time of 180 min is displayed on the upper x axes of both plots. The range for the lethal dose 90% (LD90) is displayed as the gray box with annotation.

Figure 4 shows the inhibitory effect of blue light irradiance on biofilm formation over three experiments represented in Figure 4A by CFU counts per coupon and in Figure 4B on a relative scale as survival fraction. The inoculation concentration could not be controlled but was recorded for each experiment and is displayed in the plot legends for reference. The data shows that blue light at the irradiance range used has a significant inhibitory effect on biofilm formation and there is a strong correlation to a one phase exponential decay model detailed in the methodology. A 90% reduction in biofilm formation was achieved at an irradiance range18–32 mW/cm^2^ and dose (fluence) range of 194–346 J/cm^2^. Biofilm formation was completely inhibited to below the detection limit at an irradiance of 160 mW/cm^2^ for all experiments (fluence of 1,728 J/cm^2^). For comparison, a previous study by Ferrer-Espada et al. (2020) has reported a non-statistically significant survival fraction of 0.21 (–0.68 log_10_) when 24 h established PM biofilms were exposed to a blue light fluence of 216 J/cm^2^. Our study reports statistically significant, similar, or higher levels of inhibition for the same fluence. It should be noted that our study examines blue light mediated inhibition of biofilm formation rather than inhibition of pre-established biofilms. The experiment performed with the highest inoculation concentration of 1 × 10^8^ CFU/mL showed the greatest inhibitory response to increasing irradiance. It is possible that a higher starting concentration of planktonic bacteria under irradiation will lead to a higher concentration of reactive oxygen species in the artificial urine medium (AUM) leading to an enhanced antimicrobial effect. Further experimental repeats over a range of seeding concentrations will be required to establish the effect of inoculation concentration on the inhibitory effect of blue light.

**FIGURE 5:**
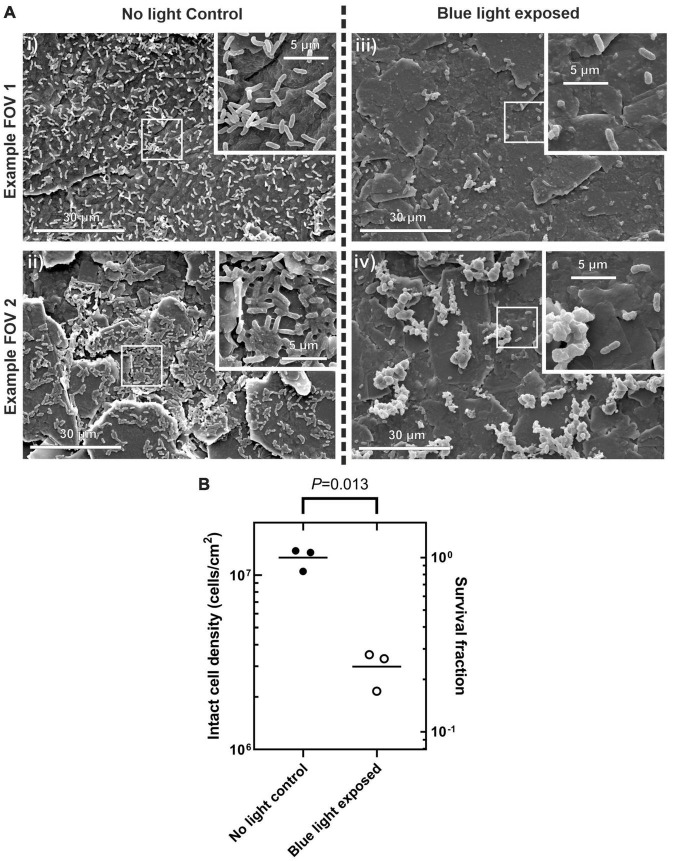
**(A)** scanning electron microscopy (SEM) micrographs of silicone coupon surface after incubation with *PM*-spiked AUM. Wide field images taken at 2,000× (i), (ii) control conditions with no blue light exposure, (iii,iv) after blue light exposure at 160 mW/cm^2^. Images inset into the top right of each frame show detail at 10,000×. **(B)** Surface cell density counted by eye for each treatment. Right hand y-axis shows survival fraction to illustrate relative difference in cell density between control and blue light treatment. Welch’s *t*-test *P*-value indicated at top.

Scanning electron microscopy was used to assess biofilm morphology and to count intact cells present on the surface for the silicone coupons not exposed to blue light with those exposed to the maximum irradiance of 160 mW/cm^2^. Figure 5 shows scanning electron microscopy (SEM) micrographs of the silicone surfaces after incubation with *PM*-spiked AUM, with and without blue light treatment. Those surfaces incubated without blue light treatment show a greater coverage of intact cells, spread more evenly as individuals. The short rod shape of the cells exhibited for both treatments indicate they are in the swimmer cell state and not in the elongated swarming state (Latta et al., 1999). Those surfaces exposed to blue light show a lower coverage of intact cells with those cells remaining mostly forming into clumps, shown in detail in Figure 5Aiv. These clumps appear to be bound and covered by extracellular polymeric substance and the cells within are stacked upon one another. It is possible that blue light irradiation from below has resulted in selection of only clumped cells on the surface. The combination of cell stacking, and the coverage of the EPS may produce a “sunscreen” effect whereby the blue light radiation is attenuated before reaching buried cells increasing survival rates of clumped cells. Further investigation of this behavior could yield insights into possible mechanisms for antimicrobial tolerance to blue light treatment.

Manual counts of intact cells, shown in Figure 5B revealed there was a significant reduction in the density of intact cells with blue light exposure with [Welch’s *t*-test, 2 tailed *t*(2) = 8.61, *P* = 0.013, 95% confidence interval of the difference in intact cell density of 0.48–1.40 × 10^7^ cells/cm^2^]. The survival fraction of intact cells was 0.24 when counted by eye on the SEM which is up to four orders of magnitude higher than the survival fraction of 0.2–1 × 10^–4^ for the equivalent irradiance when counted by CFU. This observation shows that the overall antimicrobial effect may occur on three fronts: (i) Planktonic cells are inhibited and do not attach to the surface. (ii) The actual attachment process of the planktonic cells is inhibited (iii) A large proportion of those cells that do attach to the surface then accumulate sufficient fluence to become unculturable. Further investigation will be required to determine if the intact cells on the surface remain viable but not culturable.

### Microbiological evaluation of light-guiding catheters

**FIGURE 6:**
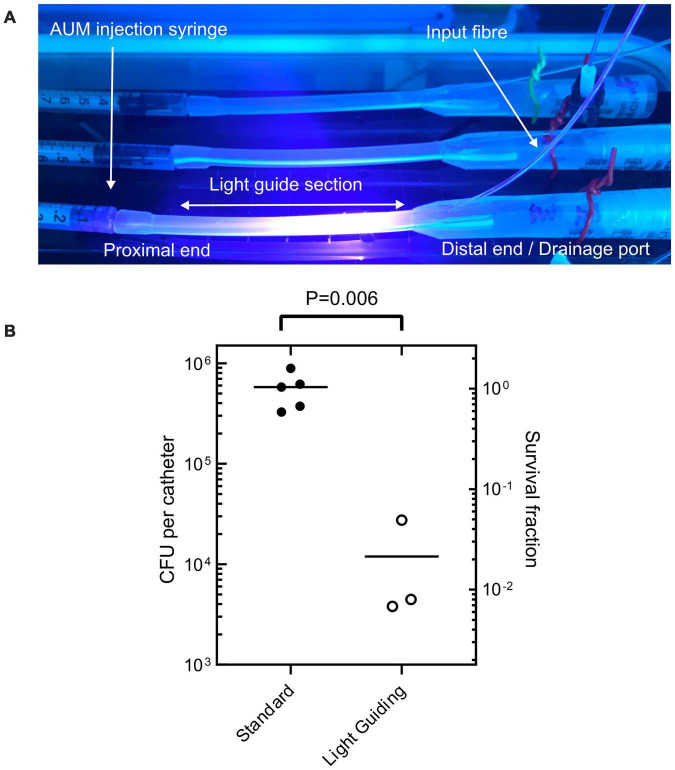
**(A)** Sections of light-guiding catheter filled with *PM*- inoculated AUM under incubation. **(B)** Colony forming units recovered from internal catheter surfaces after incubation with *PM*-spiked AUM for both standard non-light-guiding catheters and light-guiding catheters. Welch’s *t*-test *P*-value indicated at top.

The effectiveness of the LGC at inhibiting the formation of *PM* biofilms on internal catheter surfaces was tested. This experiment was designed to simulate one of the contamination scenarios shown in Figure 1 where contaminated urine in a leg bag seeds a *PM* biofilm inside the lumen of the catheter. Short sections of catheter, shown in Figure 6A, were prepared which included the drainage valve, and 4 cm of upstream catheter tubing with a single waveguide integrated in the side wall. These catheter sections were incubated with *PM*-spiked AUM and the resultant biofilm growth in the lumen was compared between LGCs and standard catheters where there was no blue light exposure.

Figure 6 shows that on average there were 5.5 × 10^5^ fewer culturable cells recovered from the LGCs compared to the standard catheters [Welchs *t*-test, 2 tailed *t*(4) = 5.39, *p* < 0.05, 95% confidence interval of the difference in cell growth = 2.7 × 105–8.3 × 10^5^ cells]. The LGCs therefore exhibited on average a 98% inhibition of PM biofilm formation on their internal surfaces. The level of inhibition is comparable to the most effective antibiotic for inhibiting PM biofilm formation which is ciprofloxacin at 93% inhibition (Wasfi et al., 2020). The average irradiance experienced by bacteria inside the catheter lumen could not be experimentally measured, however, it can be estimated using the irradiance-inhibition relationship displayed in Figure 4B. A 98% inhibition is expected for an irradiance range of 30–50 mW/cm^2^ and fluence range of 324–540 J/cm^2^. The significant inhibitory effect observed with the light guiding catheters would be expected to slow biofilm formation and significantly reduce the chance of blockage in this section of catheter over a prolonged period. These results show that the use of waveguide-based blue light irradiation in catheters presents a new avenue to target PM in addition to the main UTI-causing bacteria (Halstead et al., 2016). Future work will aim to design LGCs to produce a maximal antimicrobial effect for minimum optical input power, whilst remaining safe to use. This will include enhancing coupling efficiency and homogenizing the irradiance profile along the length of the catheter by using a terminal mirror at the end of the waveguide in addition to a varying concentration of scattering nanoparticles with waveguide length. It remains to be established if the intensities used in this study are safe for long term use inside the body (Leanse et al., 2022) however, additional masking layers of opaque silicone could be included in the catheter design to shield tissues from high levels of irradiation while still exposing the lumen to prevent biofilm growth and catheter blockage. Additionally LED pulsing can be used to reduce overall fluence while retaining antimicrobial efficacy (Gillespie et al., 2017).

## Conclusion

We have successfully demonstrated the novel concept of a LGC for the inhibition of *PM* biofilm formation. The device uses a leaky silicone waveguide to irradiate the internal surfaces of the catheter with antimicrobial blue light forming a light gate region which can prevent the ingress of biofilms up the catheter aiming to ultimately decrease the chance of catheter blockage and UTI. The antimicrobial effect of blue light on *PM* biofilm formation over a range of irradiances has been described for the first time showing an LD90 at 192–345 J/cm^2^ and total inhibition of biofilm formation at 1,700 J/cm^2^ fluence. We have described an accessible, low-cost fabrication process for light sources and LGCs using off-the-shelf components. The LGCs, while prototypes, showed sufficient optical performance to be used in antimicrobial studies. The prototype LGCs showed a 98% inhibition in biofilm formation inside the catheter lumen at an average estimated irradiance of 30–50 mW/cm^2^ (324–540 J/cm^2^ fluence) showing that the concept is highly effective and promises to be a powerful and economical antimicrobial approach to prevent catheter associated biofilm development and blockage.

## Materials and methods

### Blue light source

**FIGURE 7:**
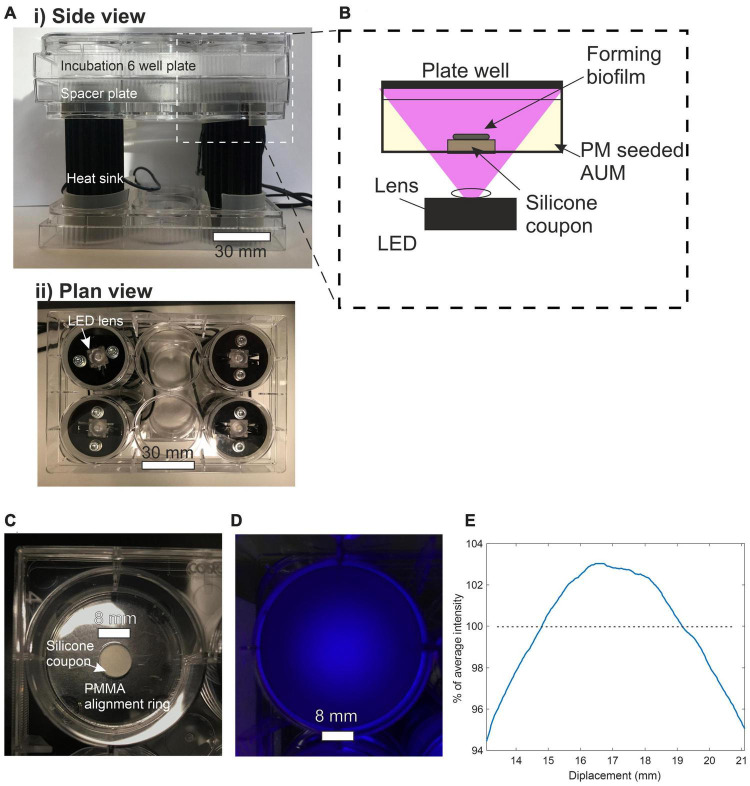
**(A)** (i) Side view of incubation plate illuminator. (ii) Plan view of incubation plate illuminator. **(B)** Side view schematic of incubation plate illuminator optical configuration. **(C)** Plan view of a single well plate containing silicone coupon. **(D)** Plan view of plate well subjected to LED illumination. **(E)** Line profile of irradiance across the center of the 8 mm diameter silicon coupon expressed a percentage of average intensity.

Figures 2, 7 show the light source used in this study which consisted of a chip-scale packaged (CSP) nominal 405 nm LED module (SZ-01-S8, Luxeonstar, US) with a nominal radiometric power of 675 mW at 500 mA and a spectral width of 10 nm at half maximum centered at a nominal wavelength of 405 nm. Each LED module was mounted with thermally conductive adhesive on a finned aluminum heat sync to allow efficient heat dissipation from the LED die. LEDs were electrically driven by a current-regulating driver (023-D-E-500 BuckPuck, LED dynamics) which allowed control of the driving current between 0 and 500 mA with a variable resistor. For free space illumination experiments, a LED lens with a 36° divergence angle was glued over the LED module (10412 LED lens, Carlco). Fiber coupled LEDs were also prepared for coupling to LGCs. Figure 2 shows the butt-coupling configuration which consisted of a modified versatile link (VL) connector mounted on a bulkhead and then optically aligned with the center of the LED emission profile and then fixed in place with bolts. This allowed for “plug and play” style reversible connection of optical fibers terminated with a VL ferrule. The optical fibers used were ESKA^®^ polymethylmethacrylate (PMMA) fibers (Mitsubishi, Japan) with a core diameter of 980 ± 59 μm and numerical aperture (NA) of 0.51 ± 0.03.

### Fabrication and optical characterization of light-guiding catheters

Silicone foley catheters (Rüsch, Teleflex, US) with an integrated balloon and inflation channel were used as a base to make the LGCs. These catheters were selected because they were made from transparent silicone with a refractive index of 1.430 at 405 nm and had a balloon inflation channel large enough to accommodate a 1 mm diameter optical fiber. To make the waveguide, high index optical silicone (LS-6946, NuSil, Avantor, US) was injected into the catheter balloon inflation channel. This silicone has a refractive index of 1.483 at a wavelength of 405 nm (NuSil, 2018) and contains fumed silica for reinforcement with a refractive index of 1.470 at 405 nm. The waveguides were butt-coupled to a 1 mm diameter optical fiber by pushing the fiber tip into the beginning of the balloon inflation channel while the silicone was still liquid. The silicone was cured at 21°C for 24 h and the free end of the optical fiber was terminated with a VL ferrule to allow coupling to the LED light source.

LGC slices were imaged on a Nikon LV100D microscope to check the quality of the waveguide core and measure its dimensions. The waveguide propagation loss was measured with the cut back method, the results of which are shown in Supplementary Figure 2. Starting with a 25–30 cm length of waveguide, the outpower was measured on a photodetector and then a length of waveguide was cut away. The output power of the remaining section of waveguide was then measured. The propagation loss for each section of waveguide removed was determined as the difference in transmitted power between successive measurements divided by the length of waveguide removed. At least two measurements were taken per waveguide over four separate waveguides and the loss measurements averaged.

The optical coupling loss between the fiber and the waveguide was measured by first measuring the output power of a length of waveguide and then measuring the output power of the fiber alone. Power measurements from the end facets of light guides and optical fibers were obtained by direct coupling to a photodetector (918-UV and 818-UV, Newport US) so that the majority of the sensor area was filled by the output spot. For high optical powers an OD3 neutral density filter was placed infront of the detector and power measurements were adjusted by a calibration factor of 1/0.87 (For further details see Supplementary Information section 1.1.1, Supplementary Figure 1, Supplementary Table 1). The coupling loss between fiber and catheter waveguide, *L*_*C*_ in dB was calculated as *L*_*C*_ = *P*_*WG*_ + *L*_*P*__l-P*F*_ where *P*_*WG*_ is the waveguide output power in dBm, *L*_*P*_ is the waveguide propagation loss in dB/cm, *l* is the waveguide length in cm and *P*_*F*_ is the fiber output power in dBm. The relative power emitted sideways from the catheter waveguide was measured by placing a photodetector (818-UV, Newport US), fitted with a 1 mm diameter aperture, at 1 cm increments along the length of the waveguide side of the catheter and recording the collected power. A double exponential decay curve was fitted to the side emission data (Supporting Information: Equations 1 and 2). The double exponential decay model describes the simultaneous higher loss propagation in the catheter bulk and the lower loss propagation in the waveguide.

### Preparation of inocula

Artificial urine medium was prepared according to the recipe developed by Brooks and Keevil (1997). Bacterial stocks were prepared by the same method for all experiments. Frozen clinical isolates of *Proteus mirabilis* (Strain number NCTC 10975) stored on cryobeads were defrosted into a 10 ml volume of tryptone soya broth (TSB) and incubated for 24 h at 37°C. 1 ml of this stock broth of *PM* was centrifuged at 750 RCF for 10 min and the pellet resuspended in the same volume of AUM.

### Antimicrobial effect of blue light irradiance on *Proteus mirabilis*

To test the antimicrobial effect of blue light on *Proteus mirabilis* over a range of irradiances a custom plate illuminator was built. The apparatus used to illuminate the cell culture plates from below is shown in Figure 7 with the LEDs supported in a holder made from empty 6 well culture plates. This format allowed the incubation plate to be stacked on top of the LEDs with each LED centered below a well and could easily fit inside an oven incubator. A single 6 well culture plate was placed between the LEDs and the incubation plates as a spacer placing the top surface of the substrate at an illumination distance of 27.4 mm. The substrates used to support biofilm growth are shown in Figure 7C and consisted of 8 mm diameter, 1.5 mm thick, disks of translucent silicone (RS components, UK) cut from a stock sheet with a biopsy punch. The silicone is polydimethylsiloxane-based, FDA-approved and equivalent to unmodified catheter silicone. Substrates were first sterilized by autoclaving and then under aseptic conditions, a single substrate disk was placed at the bottom of a flat-bottomed 6 well culture plate where it was held in the center by a PMMA retaining ring.

The average irradiance at the top surface of the substrate was measured by placing an optical power meter over the center of the well immediately above the top surface of the silicone substrate. This measurement technique accounted for transmission loss incurred as light propagates through the substrate. Transmission losses encompassing surface reflection, scattering and absorption were measured by comparing irradiances with and without a substrate in place and were recorded at 40% for a 1.5 mm thick substrate. The intensity profile of the illumination spot was imaged by photographing the substrate with white polishing paper on top. Figures 7D,E shows that the irradiance across an 8 mm diameter disc remains within 6% of the average irradiance.

Before incubation, plate wells were filled with 5 ml of either sterile AUM or inoculated AUM. Inoculated AUM was prepared at 1% dilution from stock. Five wells were incubated with inoculated AUM and illuminated with blue light over a range of irradiance. In parallel, five wells were also incubated with inoculated AUM with no subsequent blue light irradiation as positive controls. Three wells were incubated with sterile AUM and no blue light irradiation as negative controls to monitor background contamination. Plates were incubated in an oven incubator at 37°C for 180 min with the temperature of the AUM monitored with a thermometer.

After incubation, substrates were individually removed from their wells and sequentially rinsed in three 10 ml changes of sterile PBS. Individual substrates were then placed in a 20 ml Universal tube with 1 g of 2 mm diameter glass beads and 5 ml PBS. Tubes were vortexed at 3,200 rpm for 2 min to suspend the biofilms. 50 μl of the supernatant from each mechanically scrubbed substrate was then plated out on cysteine-lactose-electrolyte-deficient (CLED) medium in 50 mm diameter plates in triplicate. Some supernatant samples were diluted by a factor of 10^1^ or 10^2^ before plating out to prevent overgrowth. Plates were incubated for at least 24 h at 37°C and CFUs were counted manually. The experiment was repeated three times. The inoculation concentration for each experiment could not be controlled precisely but was measured by CFU counts.

Curves were fitted to the experimental data in GraphPad Prism version 8.4.3 using a model based on a one phase exponential decay with the formula


(1) $$N_{i}=\left(N_{0}-L\right)\times 10^{-K\,E_{e}}+L$$

where *N*_*i*_ is the CFU with blue light irradiation, *N*_*0*_ is the CFU without blue light irradiation, *L* is the curve plateau at infinite irradiance measured in CFU, *K* is the inactivation constant and *E*_*e*_ is the irradiance in mW/cm^2^. An optimal fit was achieved by minimizing the weighted sum of squared residuals with a weighting of $1/N_{i}^{2}$. The CFU data was also converted to survival fraction defined as *N*_*i*_/*N*_*o*_ to allow comparison between experiments.

### Scanning electron microscopy imaging of biofilms

Silicone coupons were incubated with AUM according to the same method as used for the irradiance studies. Control coupons not exposed to light were compared with coupons exposed to the maximum irradiance of 160 mW/cm^2^. Three repeats of each treatment were performed. Biofilms were fixed according to a protocol modified from Erlandsen et al. (2004); following incubation, coupons were gently rinsed in PBS and placed in fixative (3% glutaraldehyde, 0.15% alcian blue in 0.1 M cacodylate buffer with a pH 7.2) for 1 h followed by two 10-min rinses in 0.1 M cacodylate buffer at pH 7.2. Biofilms were dehydrated by sequentially immersing the coupons in ethanol at increasing concentrations of 30, 50, 70, 95, 100, and 100% again for 10 min at each concentration followed by critical point drying (Balzers CPD 030 critical point dryer). Coupons were mounted on SEM stubs and sputter-coated with platinum using a Quorum Q150T ES coater before being imaged on a FEI Quanta 250 SEM. Cells were counted by eye with intact cells deemed to be those which retained the cylindrical cell shape. Numbers in clumps of cells were estimated based on the size of individual cells. For each coupon 6 fields of view were analyzed at a magnification of 2000x.

### Microbiological evaluation of light-guiding catheters

The experimental configuration used to evaluate the microbiological efficacy of the LGCs is shown in Figure 6A. The cross-sectional illumination configuration inside the catheter is shown schematically in Figure 3Aii. Under aseptic conditions, LGCs were cut to length so that 5 cm of LGC extended from the drainage valve of the catheter. Control catheters without a waveguide were also cut to the same length. Catheters were filled with 1 ml of either sterile AUM (negative control) or inoculated AUM (positive controls and LGCs) by injection from a 1 ml syringe from the proximal end of the catheter. After injection the syringe was left in place and the drainage valve sealed with a sterile silicone bung. *PM* inoculated AUM was prepared at 1 × 10^9^ cells/ml (3% of stock concentration).

The configuration of the experiment was as follows. Three LGCs were incubated with inoculated AUM to test the effect of blue light irradiation. The LED output was modulated to ensure all the LGCs produced the same side emission irradiance. Five standard catheters were incubated with inoculated AUM as positive controls. Three standard catheters were incubated with sterile AUM as negative controls. All catheters were incubated in an oven incubator at 37°C for 180 min. After incubation the exterior of the catheters was sterilized by spraying with 70% ethanol and allowing to dry. Under aseptic conditions the drainage valve and the end of the catheter touching the syringe were excised leaving a 4 cm section of catheter tube. For the LGCs this is the first 4 cm of the waveguide. Each 4 cm section was cut into 1 cm sections and each of these were split down the middle to expose the internal surface of the tubing. These segments were then rinsed in three changes of 10 ml of PBS and then placed into a universal tube containing 10 ml of PBS and 3 g of 2 mm diameter glass beads. Tubes were vortexed at 3,200 rpm for 2 min to resuspend any biofilms. CFU counts were performed using the same technique used for the free space illumination experiments.

## Data availability statement

The datasets presented in this study can be found in online repositories. The names of the repository/repositories and accession number(s) can be found below: doi: 10.5258/SOTON/D2300; University of Southampton Pure repository.

## Author contributions

JB, SW, and RE: conceptualization, resources, supervision, project administration, and funding acquisition. JB, DN, CB, SW, and RE: methodology. JB: formal analysis, data curation, visualization, and writing original draft. JB, DN, and CB: investigation. All authors: writing, review, and editing.
