# Supplementary material for: A light-guiding urinary catheter for the inhibition of Proteus mirabilis biofilm formation
Source: Front Microbiol. 2022 Sep 20;13:995200. doi: 10.3389/fmicb.2022.995200 (PMC9530263; doi:10.3389/fmicb.2022.995200)
Supplement: Supplementary file 1 [file Data_Sheet_1.PDF]

Supplementary Information for: A light-guiding urinary catheter for the inhibition of *Proteus mirabilis* biofilm formation

Jonathan T. Butement[1], Daniel J. Noel[2], Catherine Bryant[2], Sandra Wilks[3] and Robert Eason[1]

[1] *Optoelectronics Research Centre, University of Southampton, UK*

[2] *School of Biological Sciences, University of Southampton, UK*

[3] *School of Health Sciences, University of Southampton, UK*

1 Materials and methods

1.1.1 Angular response of photodiode

Characterization of the angular response of a Newport 918-UV photodiode power metre. The detector was used to measure the power from a collimated 532 nm laser beam 5 mm in diameter. The power was recorded for incident light angles between 0-40 ° at 5 ° increments. The measurements were repeated 3 times. Measurements were taken with the bare detector and with an OD3 filter in place.

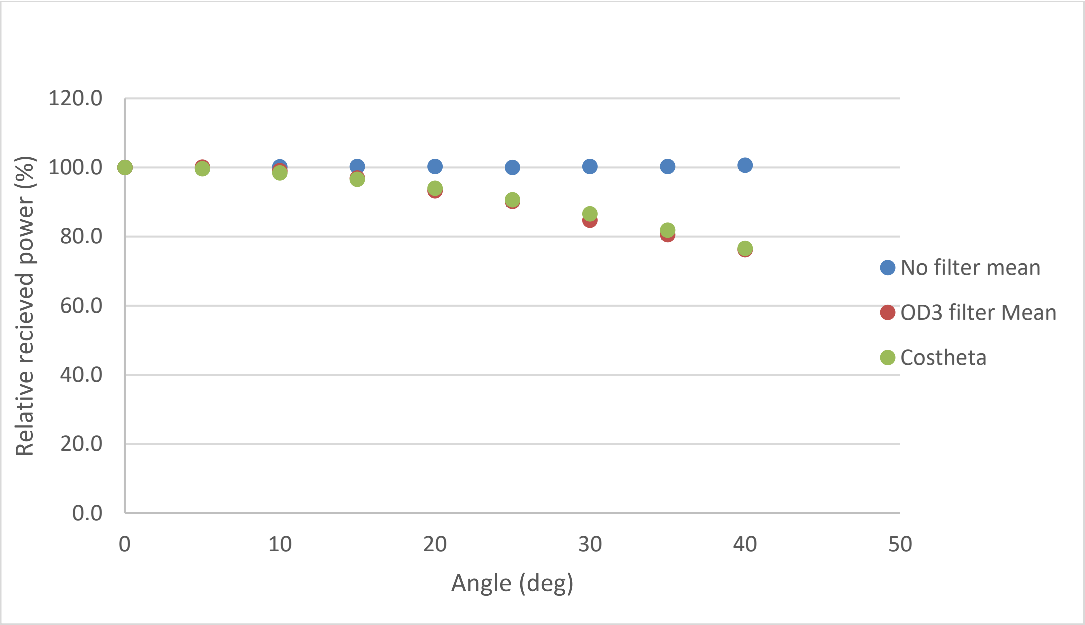

Figure S1: Response of photodiode power meter to incident light angle with and without OD3 filter in place.

1.1.2 Direct measurement of optical fibre output power.

Power measurements from the end facets of light guides and optical fibres were obtained by direct coupling to a photodetector (918-UV, Newport US) so that the majority of the sensor area was filled by the output spot. For high power measurements an OD3 neutral density filter was placed in front of the detector. The slight divergence of light from the fibre induces an under-measurement error as wider-angle rays have an increased path length through the neutral density filter. The correction factor was experimentally ascertained by comparing direct power measurements to power measurements using an integrating sphere which is typically used for divergent sources. The integrating sphere used was a Labsphere 5.3 inch Spectralon integrating sphere with a sphere multiplication factor of 53.48, fitted with a calibrated optical power meter (Newport 818-UV). Five power measurements were taken of emission from a 1mm PMMA optical fibre, interchanging between the direct measurement technique and using an integrating sphere. Supplementary Table 1 shows that on average the direct measurement technique under measures optical power from the fibres by a factor of 0.87±0.03. This correction factor was used to adjust all figures for absolute power emitted from optical fibres quoted in the main manuscript.

Supplementary Table 1: Comparison between power measurement techniques for the measurement of optical fibre output power.

	Direct 918 OD3 Power (mW)	Integration Sphere Power (No multiplication) (uW)	Integration Sphere Power (After multiplication) (mW)	Correction Factor
**1**	29.22	668.3	35.74	0.82

**2**	31.62	668.8	35.77	0.88
**3**	30.32	649.2	34.72	0.87
**4**	31.3	646.2	34.56	0.91
**5**	31.01	654	34.98	0.89
**Mean correction factor**	30.69		35.15	0.87
**SD**	0.95		0.57	0.03

1.1.3 Propagation loss measurements

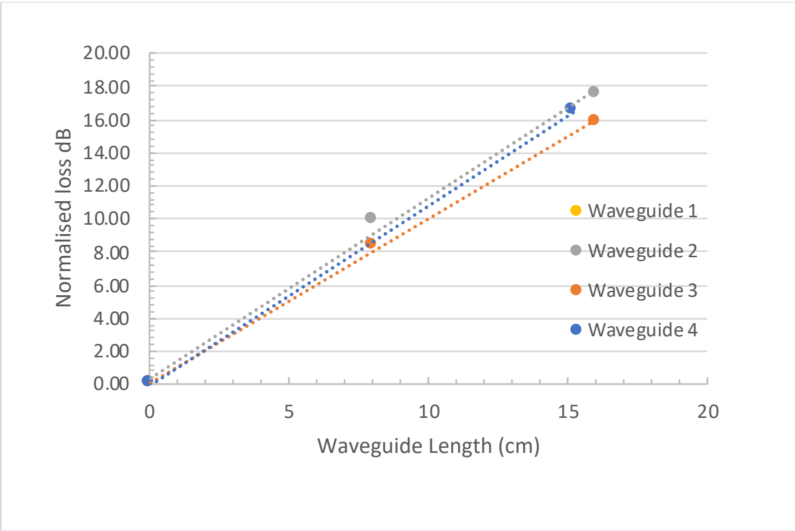

Figure S2: Waveguide propagation loss measured by cut back

1.1.4 Light guide side emission curve fitting

Curves were fit to the light guide side emission data in GraphPad Prism using a two-phase exponential decay model with the following formula.

$$P = E_1 e^{-K_1 x} + E_2 e^{-K_2 x} \qquad \text{(S1)}$$

Where E_1 and E_2 are the starting irradiance for fast and slow components of the decay in irradiance respectively and K_1 and K_2 are the rate constants for the fast and slow components of the decay respectively. The fast component of decay represents light propagation in the bulk catheter material, which exhibits a relatively higher propagation loss and the slow component of decay represents light propagation in the core of the light guide which exhibits a relatively lower propagation loss. The resulting curve of best fit was as follows.

$$P = 80.59 \times 10^{-0.530x} + 3.28 \times 10^{-0.165x} \qquad \text{(S2)}$$
